# Dose–response relationship of exercise interventions on sleep quality in patients with depression: a systematic review and meta-analysis

**DOI:** 10.3389/fpsyt.2026.1795196

**Published:** 2026-06-30

**Authors:** Ziqi Gao, Sujie Mao, Yaxin Wang, Huimin Yang, Chang Liu, Jiahe Wu, Jianxin Kang

**Affiliations:** Graduate Department, Harbin Sport University, Harbin, HeilongJiang, China

**Keywords:** depression, dose–response relationship, exercise intervention, meta-analysis, sleep quality

## Abstract

**Background:**

This meta-analysis aimed to systematically evaluate the effects of exercise interventions on sleep quality in patients with depression and to explore the dose–response relationships of key intervention parameters.

**Methods:**

A comprehensive search was conducted in PubMed, Web of Science, Embase, Scopus, and the Cochrane Library to identify randomized controlled trials (RCTs). Standardized mean differences (SMDs) and 95% confidence intervals (CIs) were calculated using a random-effects model. Subgroup and sensitivity analyses were performed to examine potential dose–response relationships.

**Results:**

A total of 17 publications, including 19 randomized controlled trial comparisons and 1,457 participants, were included in this systematic review and meta-analysis. Pooled estimates indicated that exercise interventions significantly improved sleep quality [SMD = −0.37, 95% CI: −0.48 to −0.27]. Dose–response modeling suggested that exercise doses around 312.75 MET·min/week may be associated with greater improvements [Hedges’ g = −0.51, 95% CI: −0.71 to −0.31]. Subgroup analyses suggested that mind–body exercise [SMD = −0.49, 95% CI: −0.63 to −0.35], durations of 9–12 weeks [SMD = −0.49, 95% CI: −0.74 to −0.24], fewer than two sessions per week [SMD = −0.47, 95% CI: −0.65 to −0.29], and sessions longer than 90 minutes [SMD = −0.42, 95% CI: −0.66 to −0.18] may be associated with favorable changes.

**Conclusions:**

Exercise interventions may improve sleep quality in individuals with depression, with potential benefits at low doses. These findings support individualized exercise prescriptions, but larger multicenter RCTs with long-term follow-up are needed to confirm the dose–response pattern and subgroup findings.

**Systematic Review Registration:**

https://www.crd.york.ac.uk/prospero/display_record.php?ID=CRD420251105568, identifier PROSPERO (CRD420251105568).

## Introduction

Depression is a common yet complex psychiatric disorder characterized by persistent low mood, diminished interest or pleasure, and impairments in both cognitive and physiological functioning, profoundly affecting individuals’ quality of life and social functioning (1, 2). According to the World Health Organization (WHO), approximately 332 million people worldwide suffer from depression, which has become one of the leading contributors to global disease burden and disability (3, 4). Among individuals with depression, sleep disturbance is one of the most prevalent comorbid symptoms, typically manifested as difficulty initiating or maintaining sleep and poor overall sleep quality (5), with a reported prevalence exceeding 80% (6). Such disturbances not only exacerbate depressive symptoms but also impair cognitive performance, heighten suicide risk, and reduce recovery rates (7, 8).

Sleep quality is a crucial determinant of mental health and daily functioning, encompassing multiple dimensions such as sleep duration, efficiency, depth, and subjective perception (9). It is primarily regulated through the coordinated action of neurotransmitter systems, circadian rhythm mechanisms, and emotional regulation networks (10). Previous research has shown that sleep disturbances in individuals with depression aggravate emotional dysregulation, impair executive functioning, and significantly increase the likelihood of relapse (11, 12). Therefore, improving sleep quality among patients with depression is not only essential for alleviating core symptoms but also plays a pivotal role in enhancing long-term recovery and overall quality of life.

In recent years, exercise interventions have gained increasing recognition as a promising non-pharmacological approach for managing depression. Accumulating evidence indicates that such interventions exert beneficial effects on emotional symptoms, cognitive functioning, and physical health in individuals with depression (13, 14). Various modalities—including aerobic exercise, resistance training, yoga, and Tai Chi—have been shown to effectively enhance sleep quality among this population (15, 16). The underlying mechanisms are thought to involve the promotion of melatonin secretion, improvement in autonomic nervous system balance, and enhancement of emotional regulation capacities (17, 18). However, most existing studies are limited by small sample sizes and methodological heterogeneity. The comparative efficacy of different exercise modalities, intensities, frequencies, and intervention durations remains unclear, and systematic evidence regarding their dose–response relationship is still lacking.

Khazaie et al. (19) included 10 randomized controlled trials and specifically evaluated the effects of physical activity on sleep quality in patients with major depressive disorder (MDD). Their findings indicated that physical activity improved sleep quality in individuals with MDD, with approximately 150 minutes of moderate-intensity exercise per week appearing to provide clinical benefits. However, that study primarily focused on the overall effectiveness of exercise interventions and did not standardize exercise intensity, frequency, session duration, and intervention length into a unified MET·min/week metric, nor did it construct a dose–response model to identify an optimal exercise dose.

Similarly, Xie et al. (20) conducted a systematic review and meta-analysis of 22 randomized controlled trials examining the effects of exercise on sleep quality and insomnia in adults. Their results demonstrated that exercise significantly improved subjective sleep quality, insomnia severity, and daytime sleepiness. In addition, subgroup analyses were performed according to exercise type, intervention duration, age, and sex. Nevertheless, the study population consisted of generally healthy adults, and studies involving depression and other pathological conditions were explicitly excluded.

Accordingly, the present study conducted a systematic review and meta-analysis to comprehensively evaluate the effects of exercise interventions on sleep quality in individuals with depression. Specifically, this study sought to quantify the effect sizes associated with various exercise modalities and to further explore the moderating roles of key prescription parameters—including intervention frequency, duration, and total intervention period—within a dose–response framework. Additionally, between-study heterogeneity and its potential sources were examined. The findings aim to provide an evidence-based foundation for the development of individualized exercise prescriptions and precision rehabilitation strategies for patients with depression.

## Methods

This systematic review and meta-analysis was conducted in accordance with the Preferred Reporting Items for Systematic Reviews and Meta-Analyses (PRISMA) Statement (21). The study protocol was prospectively registered in the PROSPERO database (Registration No. CRD420251105568).

### Search strategy

A comprehensive literature search was performed across five electronic databases—PubMed, Scopus, Web of Science, Embase, and the Cochrane Library—from their inception to May 25, 2026. The search was restricted to studies published in English and limited to randomized controlled trials (RCTs). Both Medical Subject Headings (MeSH) terms and free-text keywords were employed to ensure search sensitivity and specificity. The PubMed search strategy was as follows:(“Exercise”[Mesh] OR “Physical Activity”[Mesh] OR “Aerobic Exercise”[Mesh] OR “Resistance Training”[Mesh] OR “Yoga”[Mesh] OR “Tai Chi”[Mesh]) AND (“Depression”[Mesh] OR “Depressive Disorder”[Mesh] OR “Major Depressive Disorder”[Mesh]) AND (“Sleep Quality”[Mesh] OR “Sleep”[Mesh] OR “Pittsburgh Sleep Quality Index”[Mesh] OR “Polysomnography”[Mesh] OR “Insomnia”[Mesh] OR “Sleep Diary”[Mesh]).Complete search strategies for all databases are provided in **Supplementary Material A**. The literature search and study selection were independently conducted by two reviewers under a double-blind protocol. Any discrepancies were resolved through consultation with a third reviewer.

### Inclusion criteria

1. Participants were considered to have depression if they met at least one of the following criteria: a diagnosis of depressive disorder established by a clinician or through a structured/semi-structured interview according to the DSM or ICD; or scores exceeding a clinically meaningful threshold for depressive symptoms on a validated depression assessment scale, as predefined in the original study. Studies were excluded if depression was assessed solely as an outcome measure, if only non-specific psychological distress was reported, or if no formal depression diagnosis or clearly defined scale-based eligibility threshold was provided.
2. The article was published in English.
3. The study design was a randomized controlled trial (RCT).
4. The intervention consisted of any form of structured, exercise-based physical activity.
5. The control group did not receive any exercise intervention or received only non-exercise interventions such as standard health education or psychological counseling.
6. The study included at least one quantitative measure of sleep quality as an outcome variable—such as the Pittsburgh Sleep Quality Index (PSQI), Total Sleep Time (TST), Sleep Quality Questionnaire (PSQ), Visual Sleep Scale (VSH), or Insomnia Severity Index (ISI)—to assess the effects of exercise interventions on sleep quality.

### Exclusion criteria

1. Animal experiments or non-human studies.
2. Duplicate publications, studies with low methodological quality, or those presenting a high risk of bias.
3. Studies for which the full text was unavailable or from which relevant sleep quality data could not be extracted.
4. Studies that did not employ validated instruments to assess sleep quality as an outcome measure.
5. Non-original studies, including reviews, systematic reviews, meta-analyses, conference abstracts, expert commentaries, or secondary analyses of existing data.
6. Participants were required to be explicitly identified in the original studies as having a diagnosis of depression or clinically significant depressive symptoms. Studies were excluded if participants had severe physical illnesses that could substantially limit exercise participation or directly affect sleep outcomes. Such conditions included, but were not limited to, unstable cardiovascular disease, severe neurological disorders, active malignancy, severe musculoskeletal disorders, and other medical conditions with clear contraindications to exercise. Studies involving participants with other major psychiatric disorders, such as schizophrenia or bipolar disorder, were also excluded.

### Study selection and data extraction

Two reviewers independently conducted the study selection, data extraction, and quality assessment, with disagreements resolved by consultation with a third reviewer. All retrieved records were imported into EndNote 21, and duplicate entries were removed. Studies were screened according to the predefined inclusion and exclusion criteria: titles and abstracts were reviewed for initial screening, followed by full-text assessments to confirm eligibility. The extracted data included:

1. Study characteristics—first author, year of publication, and journal source;
2. Participant and intervention characteristics—sample size, mean age, intervention duration, session length, and intervention frequency for both experimental and control groups;
3. Outcome measures—quantitative sleep-related indicators such as the PSQI, TST, PSQ, VSH and ISI.

When essential data were missing or incomplete, the reviewers attempted to contact the corresponding authors via email to obtain the necessary information.

### Quality assessment

The risk of bias in randomized controlled trials was assessed using the Cochrane Risk of Bias tool, version 2 (RoB 2; Cochrane Collaboration, London, UK). RoB 2 evaluates bias across 5 key domains: the randomization process, deviations from intended interventions, missing outcome data, measurement of the outcome, and selection of the reported result. Each domain was judged as “low risk of bias,” “some concerns,” or “high risk of bias.” Assessments were conducted independently by 2 researchers, and any disagreements were resolved through discussion until consensus was reached.

### Statistical analysis

All statistical analyses were conducted using Stata version 18.0. As all outcome variables were continuous, the weighted mean difference (WMD) was calculated when studies used identical measurement instruments, whereas the standardized mean difference (SMD) was applied when different tools were employed. Both effect estimates were presented with their corresponding 95% confidence intervals (CIs).

Between-study heterogeneity was quantitatively assessed using the p-value and the I² statistic, which indicates the proportion of total variation due to heterogeneity rather than chance. A p-value > 0.10 was considered indicative of negligible heterogeneity, whereas p ≤ 0.10 denoted significant heterogeneity. I² values were interpreted as follows: ≤ 50% indicated low heterogeneity (fixed-effects model applied), 50%–75% represented moderate heterogeneity, and > 75% reflected substantial heterogeneity (random-effects model applied). When significant heterogeneity was observed, subgroup analyses were conducted to explore potential sources of variation. Statistical significance was defined as p < 0.05.

Publication bias was assessed visually using funnel plots, and leave-one-out sensitivity analyses were performed to examine the robustness of the pooled estimates.

To explore the dose–response relationship between exercise dosage and intervention effects, exercise doses were standardized according to metabolic equivalent (MET) values (22). Exercise intensity, frequency, and duration reported in each study were converted into a unified metric of weekly total exercise volume (MET·min/week). To explore the dose–response relationship between exercise dosage and intervention effects, exercise doses were standardized using metabolic equivalent (MET) values (22). Exercise intensity, frequency, and duration reported in each study were converted into a unified measure of weekly exercise volume (MET·min/week). Subsequently, restricted cubic spline (RCS) models within a random-effects framework were applied to examine the dose–response relationship between exercise dose and improvements in sleep quality. Following commonly recommended methodological practices for dose–response meta-analyses, four knots were placed at the 5th, 35th, 65th, and 95th percentiles of the exercise-dose distribution (23). Nonlinearity was assessed by comparing the spline model with a linear model, with statistical significance defined as P < 0.05 (24).

## Results

### Literature search results

**Figure 1:**
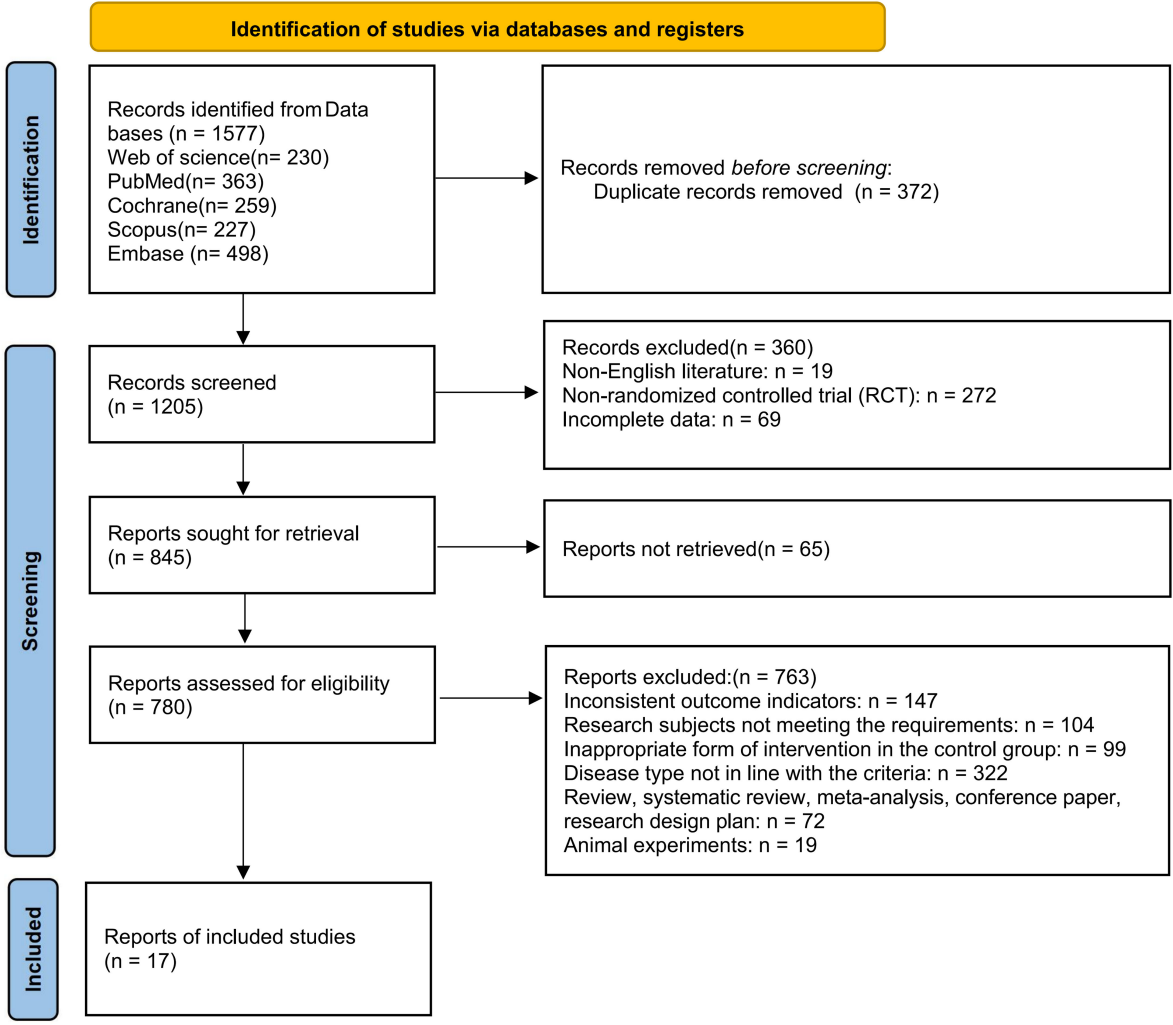
PRISMA flow diagram of study selection.

A total of 1577 records were initially identified through database searches in PubMed, Embase, Scopus, Web of Science, and the Cochrane Library. The detailed selection process is illustrated in **Figure 1**. After removing duplicates, screening titles and abstracts, and assessing the full texts for eligibility, 17 publications were finally included, yielding 19 independent randomized controlled trial comparisons for the meta-analysis (25–41).

### Characteristics of included studies

A total of 17 publications (19 randomized controlled trials) (25–41) comprising 1457 participants were included in this meta-analysis. The participants represented a broad age range, from adolescents to older adults. One study included adolescent participants aged approximately 14–18 years (41). Several studies involved young or younger adult populations aged approximately 20–39 years (29, 30, 35, 37, 39). Other studies focused on middle-aged adults aged approximately 40–59 years (25, 26, 28, 31, 33, 40). Studies involving older adults aged 60 years and above included (27, 32, 34, 36, 38). Regarding intervention modalities, aerobic exercise was examined in several studies (28, 30, 31, 35, 38, 41). Mind–body exercise was the most frequently investigated modality, including Tai Chi, yoga, dance, and other mind–body-based interventions (25–27, 29, 32–34, 37, 39, 40). Resistance exercise was examined in two studies (35, 36).

Specifically, the included studies covered multiple geographic regions, including Asia, North America, Europe, and Oceania. Studies conducted in Asia originated from mainland China (35, 41), Hong Kong, China (25, 26, 28), Taiwan, China (27), South Korea (38), and Iran (36). North American studies were conducted in the United States (29, 34, 37, 40) and Canada (30). European studies were conducted in Switzerland (31), Türkiye (32), and Sweden (33). Studies from Oceania were conducted in Australia (39). Overall, the included studies demonstrated a certain degree of geographic and cultural diversity.

With respect to outcome assessment, the PSQI was the most commonly employed instrument. Other studies utilized the TST, PSQ, VSH and ISI to evaluate sleep-related outcomes. Among the included randomized controlled trial comparisons, 16 comparisons reported extractable information on participant sex distribution, whereas 3 comparisons did not provide the specific numbers of male and female participants (35, 38). Among the studies reporting sex-related data, a total of 304 male participants and 848 female participants were included. Detailed sex distribution data are provided in **Table 1**.

**Table 1:** Characteristics of included studies.

First author	Country	Experimental group	Control group	Males/ females (n)	Age (years)	Motion type	Intervention duration (weeks)	Session duration (min/session)	Frequency (sessions/week)	Outcome indicator	Depression criteria category
Park (38)	South Korea	12	12	NR	75.20 ± 6.25	Aerobic exercise	8	50	3	PSQI	①
Gourgouvelis (30)	Canada	8	8	4/12	39.31 ± 7.02	Aerobic exercise	8	150	2	PSQI	①+②
Imboden (31)	Switzerland	22	19	22/20	45.0 ± 10.5	Aerobic exercise	6	50	3	PSQI	①+②
Zhang (41)	China	66	69	75/63	14.4 ± 2.2	Aerobic exercise	16	60	5	PSQI	①+②
Cheung (28)	Hong Kong, China	17	17	7/27	47.8 ± 11.8	Aerobic exercise	12	60	1	PSQI	①+②
Liu (2026a)	China	77	79	NR	41.54 ± 13.52	Aerobic exercise	6	45	3	PSQI	②
Lavretsky (34)	United States	33	35	28/45	69.1 ± 7.0	Mind-body exercise	10	120	1	PSQI	②
Chan (25)	Hong Kong, China	16	16	11/40	46.4 ± 0.98	Mind-body exercise	10	90	1	TST	①+②
Chen (27)	Taiwan, China	62	66	35/93	69.20 ± 6.23	Mind-body exercise	24	70	3	PSQ	②
Field (29)	United States	37	38	0/92	26.6 ± 5.5	Mind-body exercise	12	20	1	VSH	①
Kerkez (32)	Turkey	58	56	43/71	70.89 ± 4.23	Mind-body exercise	6	40	3	PSQI	③
Chan (26)	Hong Kong, China	92	93	46/139	55.28 ± 9.83	Mind-body exercise	8	180	1	PSQI	②
Köhn (33)	Sweden	8	19	3/34	52.0 ± 15.0	Mind-body exercise	12	60	1	ISI	②
Oretzky (37)	United States	29	24	9/44	25.6 ± 3.22	Mind-body exercise	5	60	2	PSQI	②
Pinniger (2013a)	Australia	12	23	2/22	39.5 ± 5.0	Mind-body exercise	8	90	1	ISI	③
Pinniger (2013b)	Australia	18	23	5/59	39.5 ± 5.0	Mind-body exercise	8	90	1	ISI	①+②
Schuver (40)	United States	18	16	0/40	45.55 ± 12.30	Mind-body exercise	12	60	2	PSQI	①+②
Motamedi (36)	Iran	30	31	14/47	64.00 ± 4.35	Resistance exercise	16	30	5	PSQI	③
Liu (2026b)	China	78	79	NR	41.54 ± 13.52	Resistance exercise	6	45	3	PSQI	②

PSQI, Pittsburgh Sleep Quality Index; ISI, Insomnia Severity Index; TST, Total Sleep Time; PSQ, Sleep Quality Questionnaire; VSH, Verran and Snyder-Halpern Sleep Scale. Depression criteria category:① Clinical diagnosis; ② Scale-based threshold; ③ Self-reported/unclear criterion.

### Quality assessment of included studies

**Figure 2:**
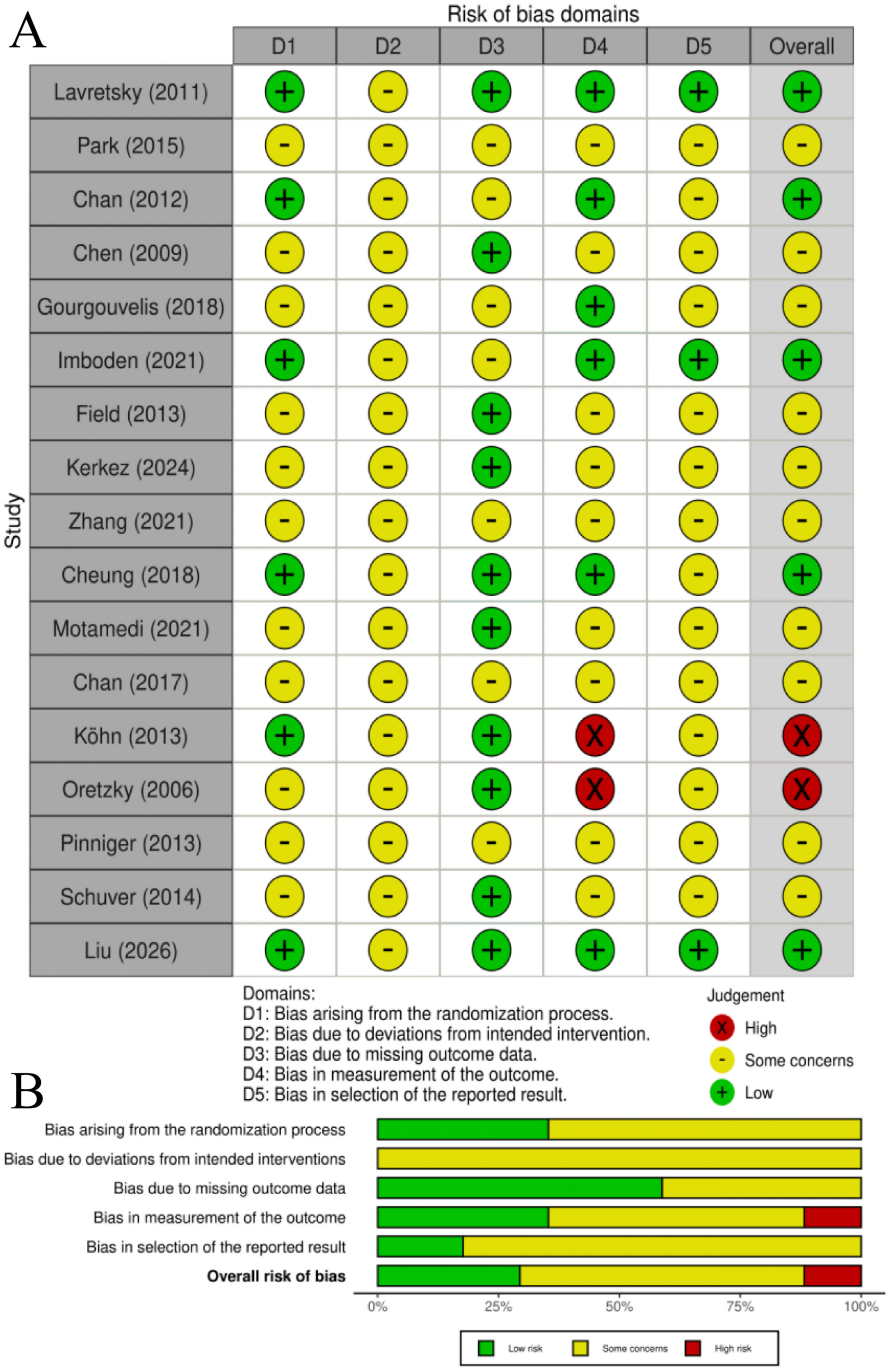
**(A)** Distribution of methodological quality; **(B)** Risk-of-bias graph and summary.

Methodological quality was assessed using the Cochrane Risk of Bias tool, version 2 (RoB 2) (**Figure 2**). In the domains of the randomization process, missing outcome data, and selection of the reported result, most studies did not exhibit substantial methodological concerns. In the domain of deviations from intended interventions, several studies showed a certain degree of uncertainty. In the outcome measurement domain, some studies raised methodological concerns, with comparatively greater risk of bias observed in this domain (33, 37). Overall, the included studies were of generally good methodological quality, although some uncertainty remained regarding outcome measurement and the selection of reported results (**Figure 2**).

### Meta-analysis results

#### Effects of exercise interventions on sleep quality

**Figure 3:**
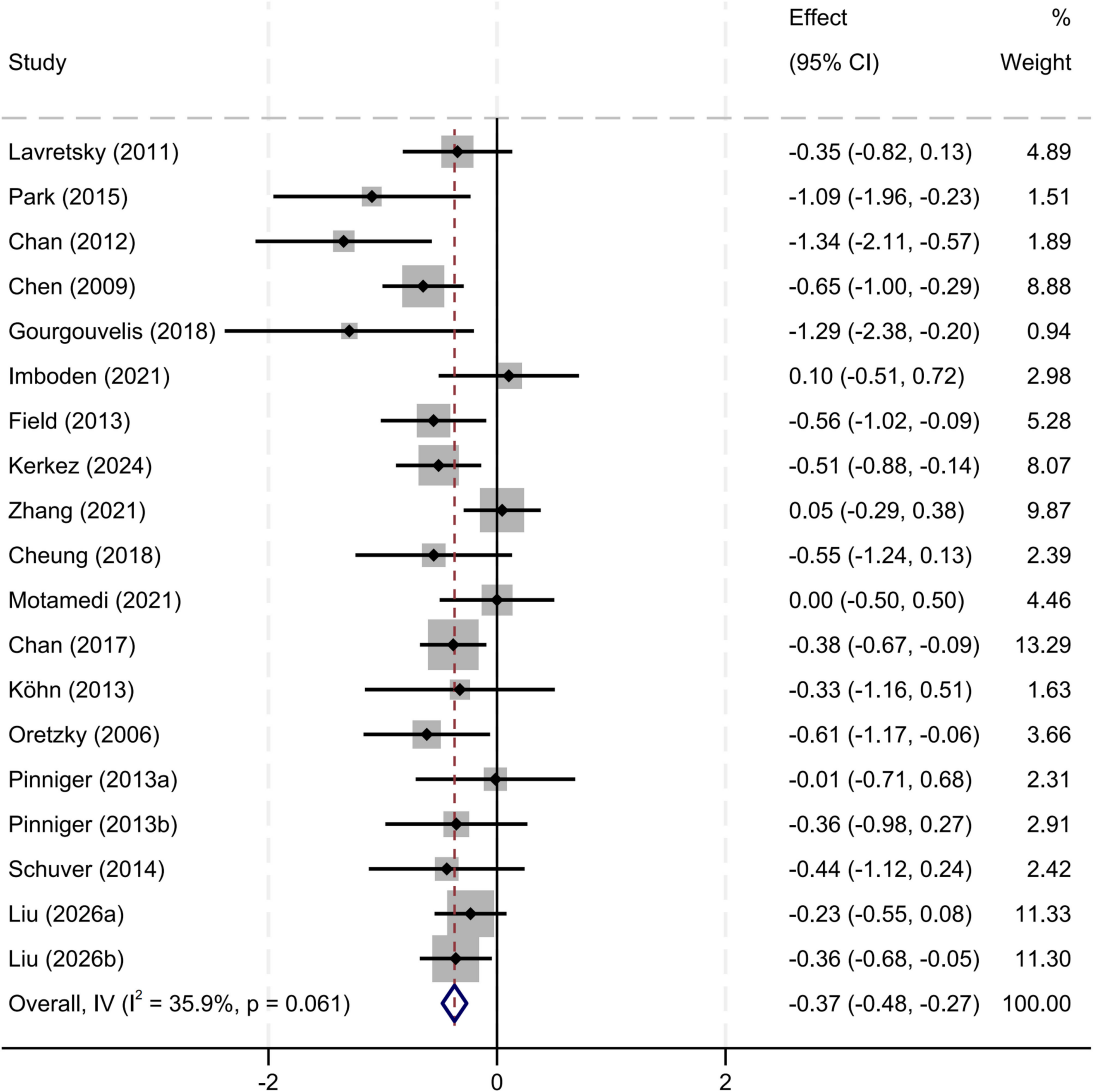
Forest plot of the effects of exercise interventions on sleep quality.

A total of 19 randomized controlled trial comparisons were included to evaluate the effects of exercise interventions on sleep quality among individuals with depression (**Figure 3**). The heterogeneity across studies was low to moderate and did not reach statistical significance (I² = 35.9%, p = 0.061); therefore, a fixed-effect model was applied.

The pooled analysis showed that exercise interventions produced a significant improvement in sleep quality compared with control conditions, with an overall effect size of SMD = −0.37 (95% CI: −0.48 to −0.27).

#### Publication bias

**Figure 4:**
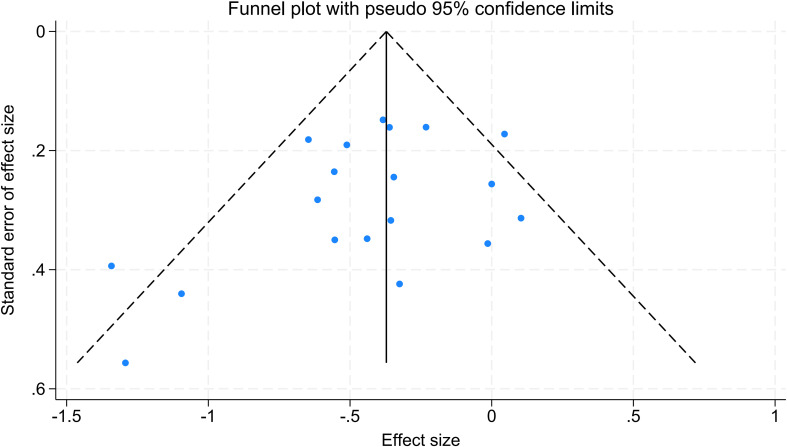
Funnel plot assessing publication bias in the effects of exercise interventions on sleep quality.

Publication bias was assessed visually using a funnel plot (**Figure 4**). The distribution of study points appeared largely symmetrical on both sides of the central axis, with only a few small-sample studies deviating slightly from symmetry. Overall, the funnel plot showed no substantial asymmetry, suggesting a low risk of publication bias in the present meta-analysis.

#### Dose–response relationship analysis

**Figure 5:**
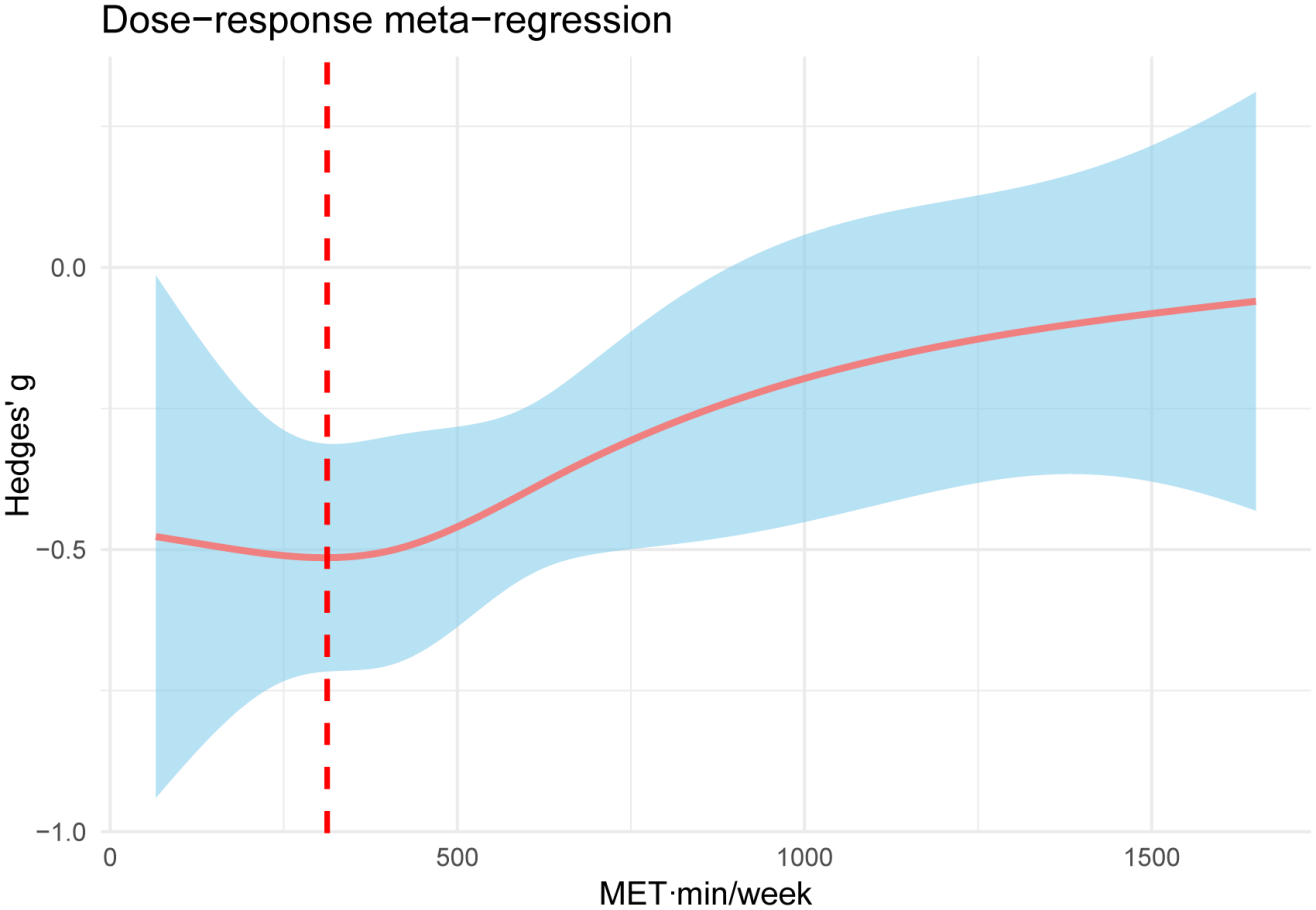
Nonlinear dose–response relationship between exercise interventions and sleep quality.

**Figure 5** illustrates the nonlinear dose–response relationship between exercise dosage and sleep quality in patients with depression, modeled using a restricted cubic spline (RCS) approach with four knots placed at the 5th, 35th, 65th, and 95th percentiles of the exercise-dose distribution. To enhance the robustness of estimates derived from studies with relatively small sample sizes, Hedges’ g was adopted as the effect size metric in the dose–response analysis. The test for nonlinearity was statistically significant (P < 0.001), indicating a significant nonlinear dose–response association between exercise dose and sleep quality.

The analysis showed that the greatest estimated improvement in sleep quality occurred at approximately 312.75 MET·min/week, corresponding to a Hedges’ g of −0.514 (95% CI: −0.716 to −0.313). Within the lower-dose range (0–300 MET·min/week), the magnitude of improvement increased progressively with increasing exercise dose. Beyond approximately 500 MET·min/week, the beneficial effect gradually diminished, and the curve tended to plateau at higher exercise doses. When the weekly exercise dose exceeded 1,000 MET·min/week, the estimated effect size remained relatively stable, fluctuating between 0 and −0.23.

#### Subgroup analyses

##### Effects of exercise type on sleep quality in patients with depression

**Figure 6:**
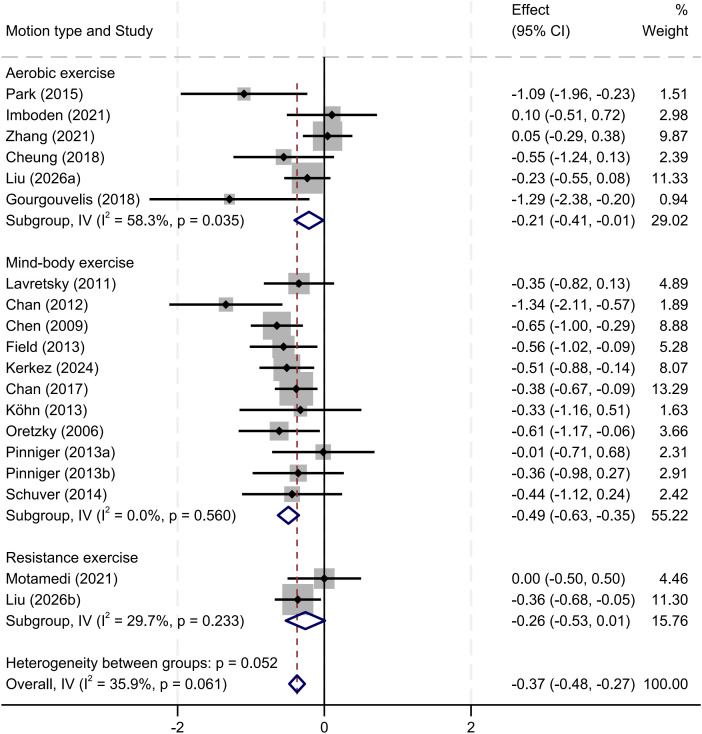
Subgroup analysis of the effects of different exercise types on sleep quality in patients with depression.

Subgroup analyses were conducted according to exercise modality (**Figure 6**). The results showed that aerobic exercise significantly improved sleep quality in patients with depression, with a pooled effect size of SMD = −0.21 (95% CI: −0.41 to −0.01). Moderate heterogeneity was observed within this subgroup (I² = 58.3%, p = 0.035).

Mind–body exercise showed a larger and statistically significant effect, with a pooled effect size of SMD = −0.49 (95% CI: −0.63 to −0.35). In contrast, resistance exercise showed a non-significant effect on sleep quality, with a pooled effect size of SMD = −0.26 (95% CI: −0.53 to 0.01), and low heterogeneity was observed (I² = 29.7%, p = 0.233). The between-group difference across exercise modalities approached statistical significance (p = 0.052).

##### Effects of intervention duration on sleep quality

**Figure 7:**
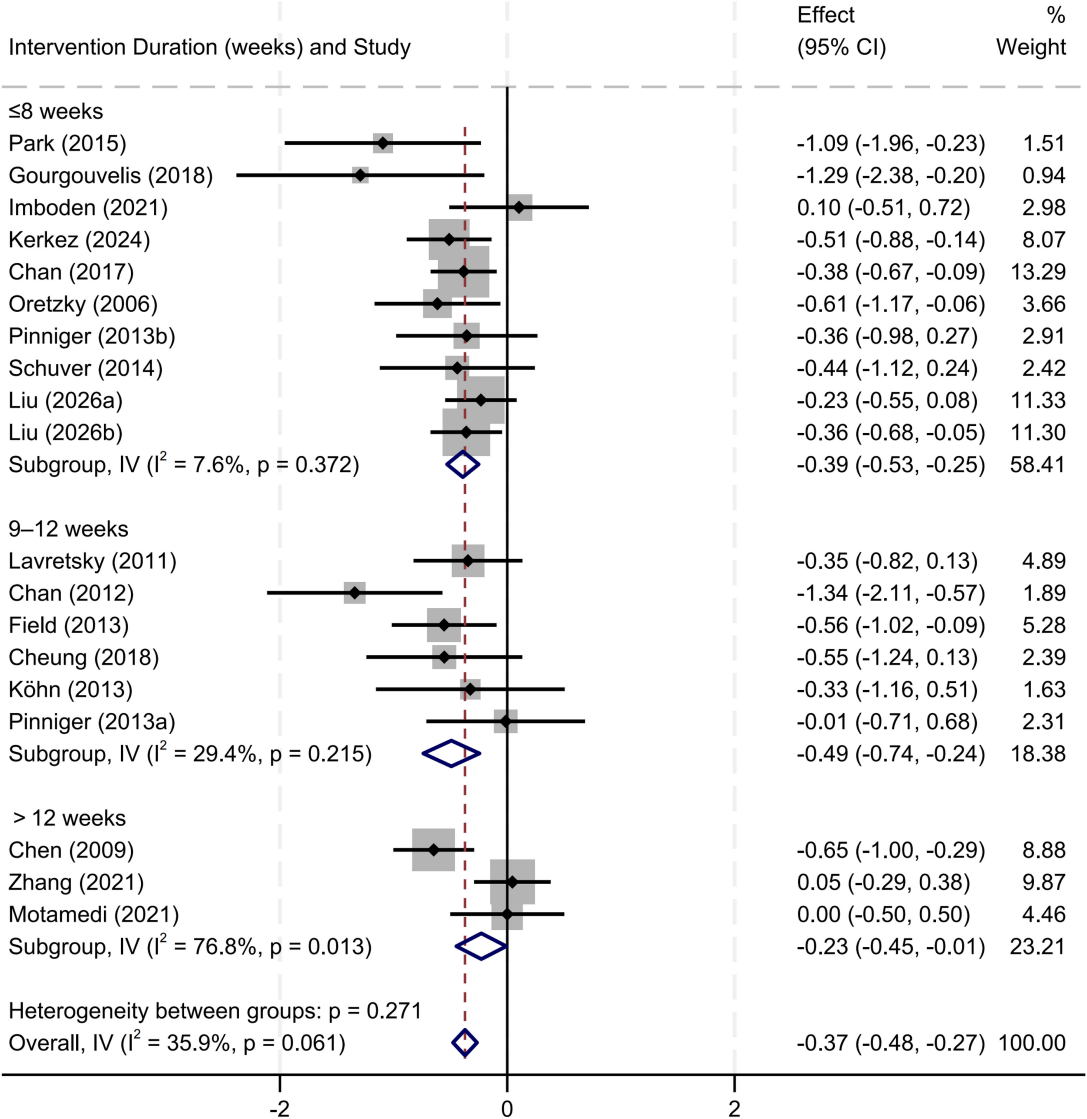
Subgroup analysis of the effects of different intervention durations on sleep quality in patients with depression.

Subgroup analyses based on intervention duration are presented in **Figure 7**. The pooled effect size for interventions lasting ≤8 weeks was SMD = −0.39 (95% CI: −0.53 to −0.25), with low heterogeneity within this subgroup (I² = 7.6%, p = 0.372). For interventions lasting 9–12 weeks, the pooled effect size was SMD = −0.49 (95% CI: −0.74 to −0.24), with low to moderate heterogeneity (I² = 29.4%, p = 0.215). Interventions lasting more than 12 weeks showed a smaller but still significant effect, with SMD = −0.23 (95% CI: −0.45 to −0.01), although substantial heterogeneity was observed in this subgroup (I² = 76.8%, p = 0.013). The between-group difference was not statistically significant (p = 0.271).

##### Effects of exercise frequency on sleep quality

**Figure 8:**
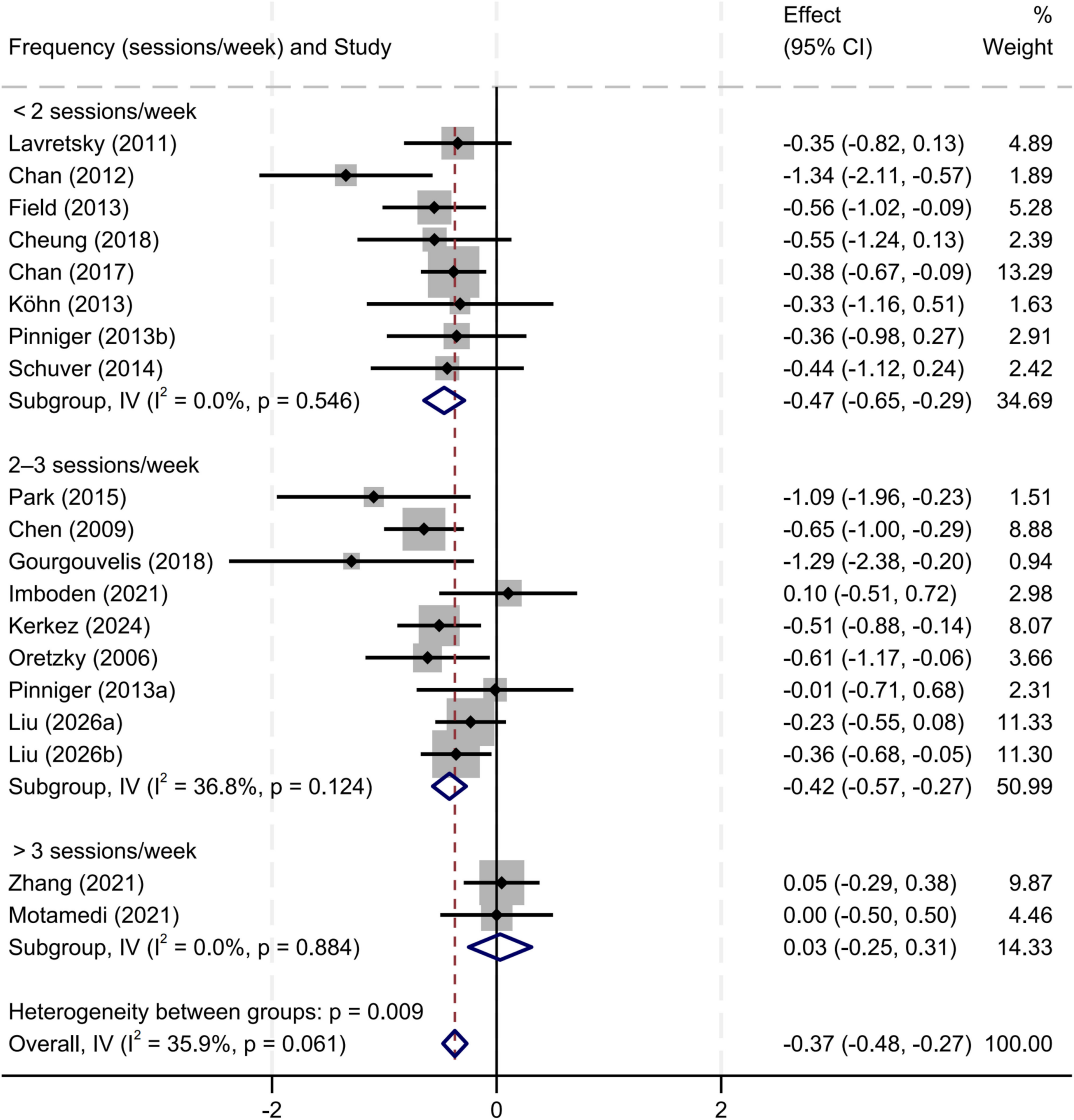
Subgroup analysis of the effects of different exercise frequencies on sleep quality in patients with depression.

Subgroup analyses by exercise frequency are shown in **Figure 8**. Interventions performed fewer than two sessions per week showed a significant effect on sleep quality, with a pooled effect size of SMD = −0.47 (95% CI: −0.65 to −0.29), and no heterogeneity was observed within this subgroup (I² = 0.0%, p = 0.546).

Interventions performed 2–3 sessions per week also significantly improved sleep quality, with a pooled effect size of SMD = −0.42 (95% CI: −0.57 to −0.27), accompanied by low to moderate heterogeneity (I² = 36.8%, p = 0.124). In contrast, interventions performed more than three sessions per week showed no significant benefit, with an effect size of SMD = 0.03 (95% CI: −0.25 to 0.31), and no heterogeneity was detected (I² = 0.0%, p = 0.884). The between-group difference was statistically significant (p = 0.009).

##### Effects of exercise session duration on sleep quality

**Figure 9:**
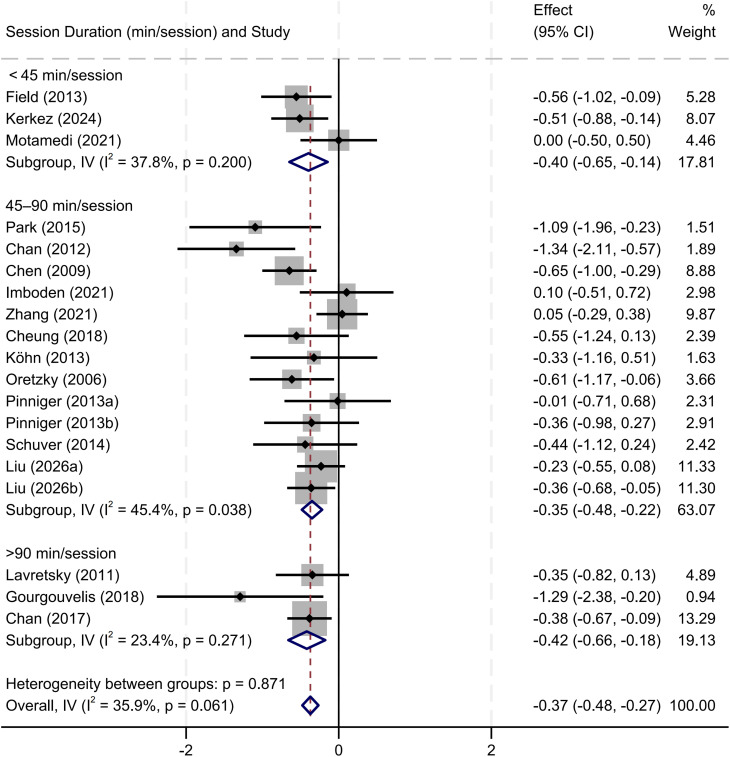
Subgroup analysis of the effects of different session lengths on sleep quality in patients with depression.

Subgroup analyses by exercise session duration are presented in **Figure 9**. The pooled effect size for sessions lasting less than 45 minutes was SMD = −0.40 (95% CI: −0.65 to −0.14), with low to moderate heterogeneity within this subgroup (I² = 37.8%, p = 0.200). For sessions lasting 45–90 minutes, the pooled effect size was SMD = −0.35 (95% CI: −0.48 to −0.22), with moderate heterogeneity (I² = 45.4%, p = 0.038). Sessions lasting more than 90 minutes also showed a significant effect, with SMD = −0.42 (95% CI: −0.66 to −0.18), and low heterogeneity was observed within this subgroup (I² = 23.4%, p = 0.271).

All three session-duration subgroups demonstrated significant improvements in sleep quality. The between-group difference was not statistically significant (p = 0.871).

### Sensitivity analysis

A leave-one-out sensitivity analysis was performed to assess the robustness of the pooled results (see **Supplementary Material B**). The re-estimated effect sizes after sequential exclusion of each individual study consistently remained within the 95% confidence interval of the overall pooled estimate (approximately -0.53 to -0.23), with only minimal fluctuation.

These findings indicate that the exclusion of any single study did not materially alter the overall effect size, thereby confirming the high stability and robustness of the meta-analytic conclusion that exercise interventions significantly improve sleep quality in patients with depression.

## Discussion

### Evidence synthesis

This meta-analysis systematically evaluated the effects of exercise interventions on sleep quality among individuals with depression and, for the first time, introduced the metric of metabolic equivalents (MET·min/week) to construct a dose–response model. This approach enabled the quantification of the nonlinear association between exercise dose and improvements in sleep quality, thereby providing an evidence-based framework for the development of personalized and precision-oriented exercise prescriptions.

The pooled results suggested that exercise interventions were associated with improvements in sleep quality among individuals with depression. Furthermore, the intervention effects appeared to vary across different exercise-dose levels. In the restricted cubic spline model, four knots were placed at the 5th, 35th, 65th, and 95th percentiles of the exercise-dose distribution. The test for nonlinearity was statistically significant (P < 0.001), indicating a nonlinear dose–response association between exercise dose and sleep quality improvement in individuals with depression.

Further dose–response modeling indicated that the optimal effect occurred at approximately 312.75 MET·min/week (Hedges’ g = -0.51). Interestingly, this optimal dose represents only about 25–50% of the weekly physical activity level recommended by the World Health Organization (600–1,200 MET·min/week) for general adults (42–44). This finding implies that, for individuals with depression, lower-dose exercise interventions may achieve a more favorable balance between efficacy, safety, and adherence (45).

These results are consistent with previous findings. Regular low-to-moderate intensity exercise significantly improved insomnia symptoms and sleep efficiency within a relatively short period (46). Low-to-moderate intensity exercise yielded superior combined effects on mood enhancement and sleep quality compared with high-intensity training (47). Supporting evidence also suggests that low-to-moderate exercise interventions exhibit advantages in safety, adherence, and long-term stability (48).

However, discrepancies exist between the present findings and studies emphasizing high-intensity exercise. High-intensity interval training (HIIT) has been shown to produce greater improvements in some patients with severe depression (49). Such differences may be attributed to variations in sample characteristics and intervention protocols.

1. Patients with severe depression often exhibit lower baseline activity levels, potentially leading to larger relative gains from higher-intensity stimulation; and
2. Several studies implemented short-term, intensive training regimens, which might have amplified short-term benefits, whereas most trials included in the present meta-analysis adopted long-term, regular exercise programs.

These discrepancies highlight the need to interpret exercise-related sleep benefits not only in terms of overall intervention effects, but also in relation to exercise dose, intensity, and population-specific characteristics. Khazaie et al. (19) focused on patients with major depressive disorder, whereas Xie et al. (20) examined general adult populations; however, neither study quantified exercise prescription parameters within a unified dose–response framework.

Building on prior meta-analyses, this study provides additional insights by quantifying exercise dose and examining dose–response associations in patients with depression. First, it focuses specifically on individuals with depression and uses sleep quality as the primary outcome, addressing a clinically important problem in this population. Second, exercise prescriptions were standardized as MET·min/week, allowing different exercise modalities, frequencies, and durations to be compared within a unified dose framework. Third, the dose–response analysis suggests that sleep benefits may not simply increase with higher exercise doses; instead, relatively low-to-moderate doses may provide stable improvements. This is particularly relevant for patients with depression, who often experience fatigue, low motivation, and poor exercise adherence.

Therefore, the main contribution of this study is not merely to confirm that exercise is beneficial, but to further explore how exercise dose may be optimized for improving sleep quality in patients with depression. These findings provide a more specific quantitative reference for future exercise prescription studies, although additional high-quality randomized controlled trials are still needed to verify differences across dose ranges.

The results of the subgroup analyses further supported the robustness and broad applicability of low-dose exercise interventions. Among different exercise prescription parameters, programs performed fewer than two times per week demonstrated the most favorable effects, whereas more frequent exercise sessions were not associated with greater improvements. This finding suggests that excessive intervention intensity or frequency may induce fatigue, reduce adherence, or increase psychological burden, thereby attenuating the therapeutic benefits of exercise. Such factors may partially account for the heterogeneity observed across studies.

In contrast, neither intervention duration nor session length significantly contributed to heterogeneity in effect sizes. Interventions with session durations longer than 90 minutes showed the largest effect estimate, while programs lasting 9 to 12 weeks yielded the most favorable improvements.

This pattern aligns with the biological mechanisms of exercise-induced neuroplasticity, which require a period of cumulative adaptation (50), indicating that structured interventions of moderate duration are particularly conducive to sleep improvement in individuals with depression (Liang.

Based on the synthesized evidence, mind–body exercise interventions, particularly those lasting 9–12 weeks, performed fewer than two times per week, and involving sessions longer than 90 minutes, may be associated with greater improvements in sleep quality among individuals with depression. When implemented under conditions ensuring safety and adherence, such regimens not only produce meaningful improvements in sleep quality but also contribute to mood enhancement and functional recovery, providing a practical and evidence-based exercise prescription for this population.

### Clinical implications of the dose–response analysis

This study underscores the pivotal role of exercise in improving sleep quality among individuals with depression, demonstrating that even low-dose interventions can elicit significant positive effects. Importantly, the findings suggest that the benefits are not confined to specific exercise modalities—both vigorous aerobic exercise and slower-paced mind–body activities such as Tai Chi or yoga showed meaningful potential to enhance sleep.

In clinical and rehabilitation practice, patients with depression often experience reduced motivation, low mood, or physical limitations, which hinder their ability to adhere to high-intensity or high-volume exercise regimens (51, 52). Under such circumstances, low-dose exercise interventions are of particular importance, as they not only maintain therapeutic efficacy but also promote adherence by allowing patients to progressively develop exercise habits within a tolerable range (53). This gradual and sustainable approach helps prevent excessive fatigue or an additional psychological burden, thereby facilitating steady improvements in sleep quality and overall health status. Moderate and regular physical activity appears to be more sustainable and safer for vulnerable populations (54). The present findings further highlight the necessity of individualized exercise prescription, emphasizing that intervention design should flexibly account for patients’ symptom severity, personal preferences, and living environments (55). Such a patient-centered approach aligns with the ongoing paradigm shift in mental health care toward personalized and precision-based interventions (56).

Although this meta-analysis provides robust evidence supporting the beneficial effects of exercise, notable research gaps remain—particularly concerning the differential effects of exercise type, intensity, and frequency under varying moderating factors. Elucidating these relationships will be crucial for optimizing clinical strategies and guiding the development of evidence-based public health interventions for depression management. Collectively, the present findings offer a practical and empirically grounded framework for clinicians and researchers engaged in mental health and rehabilitation practice.

### Strengths and limitations

This study represents the first dose–response meta-analysis to investigate the effects of exercise on sleep quality in individuals with depression. It innovatively employed metabolic equivalents (MET·min/week) to quantify exercise dosage and established a nonlinear dose–response model. A comprehensive search across multiple databases was conducted, and the findings were further validated through subgroup and sensitivity analyses, ensuring the robustness and reliability of the results. These outcomes provide an evidence-based foundation for the development of individualized exercise prescriptions in clinical and rehabilitation settings.

However, several limitations should be acknowledged. First, due to the inherent characteristics of exercise interventions, blinding of participants and researchers was challenging, leading to potential performance bias, as most participants were aware of their group assignments. Second, some subgroups had relatively small sample sizes, and the majority of included studies were conducted in high- or middle-income countries, which may limit the generalizability of the findings to broader populations. Furthermore, there was limited consistency in intervention parameters and a lack of long-term follow-up, which could influence the stability and external validity of the conclusions.

In addition, female participants constituted the majority of the samples in studies reporting sex-related characteristics, and several studies specifically enrolled female populations or samples predominantly composed of women. Therefore, the applicability of the present findings to male patients with depression should be interpreted with caution. Because most primary studies did not report sleep-quality outcomes or intervention effects separately by sex, we were unable to further evaluate potential sex-related differences in responses to exercise interventions.

Future research should aim to include larger and more diverse samples, enhance standardization of intervention protocols, and incorporate longitudinal designs to better evaluate the sustained effects of exercise on sleep outcomes in depression. Strengthening methodological rigor and expanding cross-cultural research will further enhance the practical and translational value of these findings.

## Conclusion

This meta-analysis indicates that exercise interventions may be associated with changes in sleep quality in individuals with depression. Dose–response modeling suggested that exercise doses around 312.75 MET·min/week may be related to changes in sleep quality, potentially indicating greater improvements. Subgroup observations suggest that mind–body exercise, interventions lasting 9–12 weeks, performed fewer than two times per week, and involving sessions longer than 90 minutes may be associated with more consistent changes. Nevertheless, these conclusions should be interpreted with caution due to limited sample sizes, short follow-up durations, and moderate heterogeneity across studies. Future large-scale, multicenter trials with long-term follow-up are warranted to validate these associations and further refine individualized exercise prescriptions for patients with depression.

## Data Availability

The original contributions presented in the study are included in the article/**Supplementary Material**. Further inquiries can be directed to the corresponding author.
