# Supplementary material for: Dose–response relationship of exercise interventions on sleep quality in patients with depression: a systematic review and meta-analysis
Source: Front Psychiatry. 2026 Jun 30;17:1795196. doi: 10.3389/fpsyt.2026.1795196 (PMC13364867; doi:10.3389/fpsyt.2026.1795196)
Supplement: Supplementary file 1 [file Table1.docx]

Section A

| **Detailed Search Strategies for Each Database** | |
| --- | --- |
| PubMed search: May 25, 2026 |  |
| ("Exercise"[Mesh] OR "Physical Activity"[Mesh] OR "Aerobic Exercise"[Mesh] OR "Resistance Training"[Mesh] OR "Yoga"[Mesh] OR "Tai Chi"[Mesh]) AND ("Depression"[Mesh] OR "Depressive Disorder"[Mesh] OR "Major Depressive Disorder"[Mesh]) AND ("Sleep Quality"[Mesh] OR "Sleep"[Mesh] OR "Pittsburgh Sleep Quality Index"[Mesh] OR "Polysomnography"[Mesh] OR "Insomnia"[Mesh] OR "Sleep Diary"[Mesh]) | 363 |
| Web of Science search: May 25, 2026 |  |
| TI=("Exercise" OR "Physical Activity" OR "Aerobic Exercise" OR "Resistance Training" OR "Yoga" OR "Tai Chi" OR "Exercise Intervention") AND TI=("Depression" OR "Depressive Disorder" OR "Major Depressive Disorder" OR "Depressed") AND TS=("Sleep Quality" OR "Pittsburgh Sleep Quality Index" OR "Polysomnography" OR "Insomnia" OR "Sleep Diary") | 230 |
| Cochrane Library search: May 25, 2026 |  |
| #1 "Exercise":ti,ab,kw OR "Physical Activity":ti,ab,kw OR "Aerobic Exercise":ti,ab,kw OR "Resistance Training":ti,ab,kw OR "Yoga":ti,ab,kw OR "Tai Chi":ti,ab,kw OR "Exercise Intervention":ti,ab,kw  #2 "Depression":ti OR "Depressive Disorder":ti OR "Major Depressive Disorder":ti OR "Depressed":ti  #3 "Sleep Quality":ti,ab,kw OR "Pittsburgh Sleep Quality Index":ti,ab,kw OR "Polysomnography":ti,ab,kw OR "Insomnia":ti,ab,kw OR "Sleep Diary":ti,ab,kw  #4 #1 AND #2 AND #3 | 259 |
| Embase search: May 25, 2026 |  |
| ('Exercise':ti,ab,kw OR 'Physical Activity':ti,ab,kw OR 'Aerobic Exercise':ti,ab,kw OR 'Resistance Training':ti,ab,kw OR 'Yoga':ti,ab,kw OR 'Tai Chi':ti,ab,kw OR 'Exercise Intervention':ti,ab,kw)  AND  ('Depression':ti OR 'Depressive Disorder':ti OR 'Major Depressive Disorder':ti OR 'Depressed':ti)  AND  ('Sleep Quality':ti,ab,kw OR 'Pittsburgh Sleep Quality Index':ti,ab,kw OR 'Polysomnography':ti,ab,kw OR 'Insomnia':ti,ab,kw OR 'Sleep Diary':ti,ab,kw) | 498 |
| Scopus search: May 25, 2026 |  |
| TITLE("Exercise" OR "Physical Activity" OR "Aerobic Exercise" OR "Resistance Training" OR "Yoga" OR "Tai Chi" OR "Exercise Intervention")  AND TITLE("Depression" OR "Depressive Disorder" OR "Major Depressive Disorder" OR "Depressed")  AND TITLE-ABS-KEY("Sleep Quality" OR "Pittsburgh Sleep Quality Index" OR "Polysomnography" OR "Insomnia" OR "Sleep Diary") | 227 |

Section B


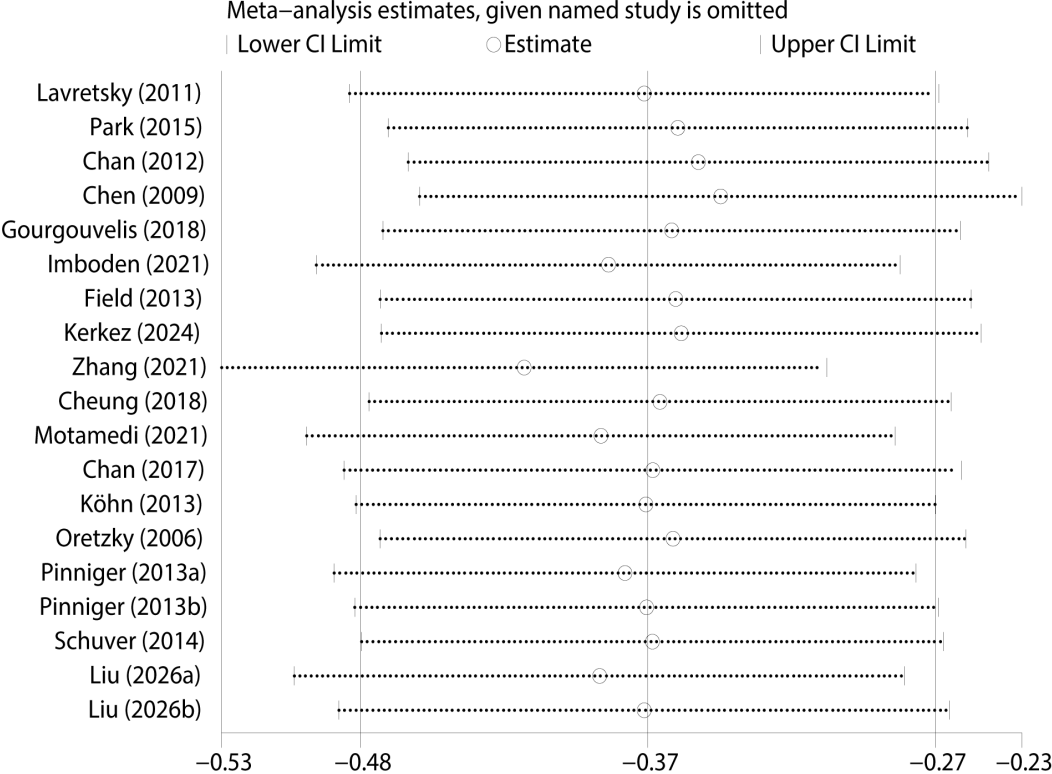


**Supplementary Figures 1**. Sensitivity analysis
